# Opportunistic CT Screening—Machine Learning Algorithm Identifies Majority of Vertebral Compression Fractures: A Cohort Study

**DOI:** 10.1002/jbm4.10778

**Published:** 2023-06-26

**Authors:** John H Page, Franklin G Moser, Marcel M Maya, Ravi Prasad, Barry D Pressman

**Affiliations:** ^1^ Center for Observational Research, Amgen Inc. Thousand Oaks CA USA; ^2^ Department of Imaging Cedars‐Sinai Medical Center Los Angeles CA USA

**Keywords:** AGING, FRACTURE PREVENTION, OSTEOPOROSIS, RADIOLOGY, SCREENING

## Abstract

Vertebral compression fractures (VCF) are common in patients older than 50 years but are often undiagnosed. Zebra Medical Imaging developed a VCF detection algorithm, with machine learning, to detect VCFs from CT images of the chest and/or abdomen/pelvis. In this study, we evaluated the diagnostic performance of the algorithm in identifying VCF. We conducted a blinded validation study to estimate the operating characteristics of the algorithm in identifying VCFs using previously completed CT scans from 1200 women and men aged 50 years and older at a tertiary‐care center. Each scan was independently evaluated by two of three neuroradiologists to identify and grade VCF. Disagreements were resolved by a senior neuroradiologist. The algorithm evaluated the CT scans in a separate workstream. The VCF algorithm was not able to evaluate CT scans for 113 participants. Of the remaining 1087 study participants, 588 (54%) were women. Median age was 73 years (range 51–102 years; interquartile range 66–81). For the 1087 algorithm‐evaluated participants, the sensitivity and specificity of the VCF algorithm in diagnosing any VCF were 0.66 (95% confidence interval [CI] 0.59–0.72) and 0.90 (95% CI 0.88–0.92), respectively, and for diagnosing moderate/severe VCF were 0.78 (95% CI 0.70–0.85) and 0.87 (95% CI 0.85–0.89), respectively. Implementing this VCF algorithm within radiology systems may help to identify patients at increased fracture risk and could support the diagnosis of osteoporosis and facilitate appropriate therapy. © 2023 Amgen, Inc. *JBMR Plus* published by Wiley Periodicals LLC on behalf of American Society for Bone and Mineral Research.

## Graphical abstract

Vertebral compression fractures (VCF) are often undiagnosed. Computer‐aided diagnosis of VCF from CT scan images of thorax or abdomen/pelvis can identify two‐thirds of patients with VCF and three‐quarters of patients with moderate to severe VCF among evaluable images, with 10% false‐positive fraction. Implementing the VCF algorithm within radiology systems may help to identify patients at increased fracture risk and could support the diagnosis of osteoporosis.

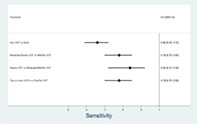

## Introduction

Osteoporosis is characterized by decreased bone mass and deterioration in bone microarchitecture^(^ ^1^, ^2^ ^)^ and is usually identified by decrements in the standard deviation scores of bone mineral density (BMD).^(^ ^3^ ^)^ Osteoporosis is associated with an increased risk of fragility fractures, including hip and vertebral fractures, but most fragility fractures occur in individuals with BMD values above the threshold used to define the disease.^(^ ^2^, ^4^, ^5^ ^)^ Fragility fractures are associated with significant morbidity and mortality, with hip fractures associated with a 1‐year mortality in excess of 20%.^(^ ^6^ ^)^

The most common fracture type associated with fragility of the bone are vertebral fractures.^(^ ^7^, ^8^ ^)^ Vertebral compression fractures (VCFs) occur when the vertebral body in the spine collapses.

Clinical presentation is quite variable, ranging from asymptomatic, height loss/kyphosis to severe pain requiring hospitalization.^(^ ^2^, ^9^ ^)^ Most vertebral fractures are clinically unrecognized, but they have importance in identifying skeletal fragility and are associated with increased risk of other fractures, including hip fracture.^(^ ^2^ ^)^ Women with pre‐existing vertebral fractures had approximately four times greater risk of subsequent vertebral fractures than those without prior fracture.^(^ ^10^ ^)^ Women with pre‐existing vertebral fractures have a 1.5‐ to 2‐fold increased risk of incident hip fracture compared with those without.^(^ ^8^ ^)^ Further, vertebral compression fractures are associated with persistent pain, as well as increased risk of progression of age‐related kyphosis (with its associated decreased pulmonary function, increased risk of gastroesophageal reflux disease [GERD], and decreased physical function).^(^ ^8^ ^)^ Finally, incident clinical vertebral fractures are associated with an initial 2‐ to 8‐fold increased age‐adjusted mortality rate.^(^ ^8^, ^11^, ^12^ ^)^

Given that many vertebral fractures are clinically silent and others present with nonspecific back pain, diagnosis is a clinical challenge. Many patients, however, receive diagnostic tests for other clinical reasons that may incidentally detect vertebral fractures. Zebra Medical Imaging algorithms are meant to assist radiologists in detecting frequently overlooked lesions. The Zebra VCF detection algorithm was developed utilizing a combination of traditional machine vision segmentation and convolutional neural network (CNN) technology^(^ ^13^ ^)^ and may be applied to CT images of the chest, abdomen, and/or pelvis. We conducted an independent and blinded validation study on previously completed CT scans of chest or abdomen/pelvis from women and men aged 50 or older, who as outpatients or inpatients, had studies at Cedars‐Sinai Medical Center in Los Angeles, CA, USA. We estimated the sensitivity, specificity, likelihood ratios, and predictive values and their associated 95% confidence intervals (CI), using the diagnosis of board‐certified neuroradiologists as the reference standard.

## Materials and Methods

### Study design and participants

Participants for this study were men and women aged 50 or older, with previously conducted CT scans of the chest or abdomen/pelvis performed as Cedars‐Sinai Medical Center within the period from 2012 to 2017, with information on age and sex. The protocol requested that consecutive CT scans be identified from the radiology record system in reverse chronologic order from June 2017 until 550 CT scans for the chest were identified from 550 unique individuals and 550 CT scans of the abdomen/pelvis were identified from 550 unique individuals. In addition, to ensure that there were enough study participants with VCF, we also sought to identify 50 CT scans of the chest and 50 CT scans of the abdomen/pelvis with previously defined verifiable VCF (based on a prior radiologist determination in the record) from the same time period. The CT scanners used to obtain the original images were of multiple types, including CT scanners made by GE, Siemens and Canon.

### Image analysis and validation

Each set of CT scan images was independently reviewed by two neuroradiologists (from a pool of three neuroradiologists, each with more than 10 years of relevant experience) to identify both the presence of compression fractures of the spine and associated grade (severity of vertebral body height loss, using the semiquantitative scale of Genant and colleagues^(^ ^14^ ^)^). The level(s) of the fractures were also determined and documented. The images were viewed by using a Carestream picture archiving communication system (PACS). The information was collected and then recorded in an independent csv file.

Scans with differences in VCF diagnoses (presence, grade, or location) were reviewed by the principal investigator (BDP) and a final diagnosis made. Study radiologists did not have access to the original CT study reports.

The Zebra Medical Vision software was installed at Cedars‐Sinai using a server disconnected from the “radiology information system,” but with access to the de‐identified CT scans. The software did not have access to participant diagnoses or to the assessments of radiologists. Further, study radiologists were also blind to the assessments provided by the Zebra Medical Vision software. The algorithm output was a csv file containing a positive/negative finding per CT study. The algorithm did not identify the location of the fracture; instead, there was a positive finding if there was a fracture identified at any vertebral level, and there was a negative finding if no fracture was identified by the algorithm at any level of the spine. The details of the algorithm were described by Bar and colleagues.^(^ ^13^ ^)^

After a full review, the study protocol was approved by the Cedars‐Sinai Institutional Review Board. Patient consent was waived by the review board.

### Data collection and protocol amendments

A separate csv file was created containing baseline information, including age and sex, and a study participant identifier. All files were exported to Amgen, where the files were merged into an analysis data set.

Data collection was planned before the performance of the index test and the reference standard. However, after initial data collection, it was discovered that the implementation team had not included the 100 CT studies with previously defined verifiable VCF. We therefore requested that the study data be augmented with an additional 200 CT studies, 100 with previously defined VCF, and the data randomly shuffled. This was done to maintain blinding.

### Statistical analysis

Based on prevalence estimates from previous studies,^(^ ^15^, ^16^ ^)^ we estimated that we needed approximately 1000 patients to determine if the Zebra VCF detection algorithm had a positive likelihood ratio above 10 (based on a sensitivity of 0.9 and specificity of 0.91) and for the 95% confidence interval to exclude a positive likelihood ratio of 8.

Analyses were conducted both at the CT study level and at the vertebral level in the spine. Results were stratified by CT study region (chest versus abdomen/pelvis). We estimated sensitivity (probability of Zebra VCF algorithm positive result conditional on presence of VCF as determined by study radiologists), specificity (probability of Zebra VCF algorithm negative result conditional on absence of VCF as determined by study radiologists), positive and negative likelihood ratios, and predictive values along with relevant 95% confidence intervals. Ninety‐five percent confidence intervals for sensitivity, specificity, and predictive values were estimated using exact binomial distributions. Likelihood ratios and their 95% CIs were estimated using log‐linear regression models. We also re‐estimated sensitivities inclusive of images that were not evaluable by the VCF algorithm.

To estimate interrater reliability of the initial evaluations of the three neuroradiologists, excluding the principal investigator (two per study participant), we used mixed‐effects linear regression models with random intercepts at the study participant level and at the radiologist level, using presence versus absence of VCF, VCF severity, or number of VCF fractures per study participant, respectively, as a continuous outcome variable. We additionally included fixed‐effects terms for the ratings of each of two radiologists (with the other modeled as the intercept). We estimated the intraclass coefficient (ICC) as the ratio of between subject variance to total variance (sum of between‐subject variance and within‐subject variance). We estimated 95% bias‐corrected with acceleration bootstrap (BCa) confidence intervals for ICCs from respective 1000 bootstrap samples for each of the three estimations.

The data analysis for this article was generated using Stata software (StataCorp, College Station, TX, USA), version 17,^(^ ^17^ ^)^ and SAS/STAT software (SAS Institute, Cary, NC, USA), version 9.4 of the SAS system for LIN X6 platform.^(^ ^18^ ^)^

## Results

### Description of sample

**Fig. 1:**
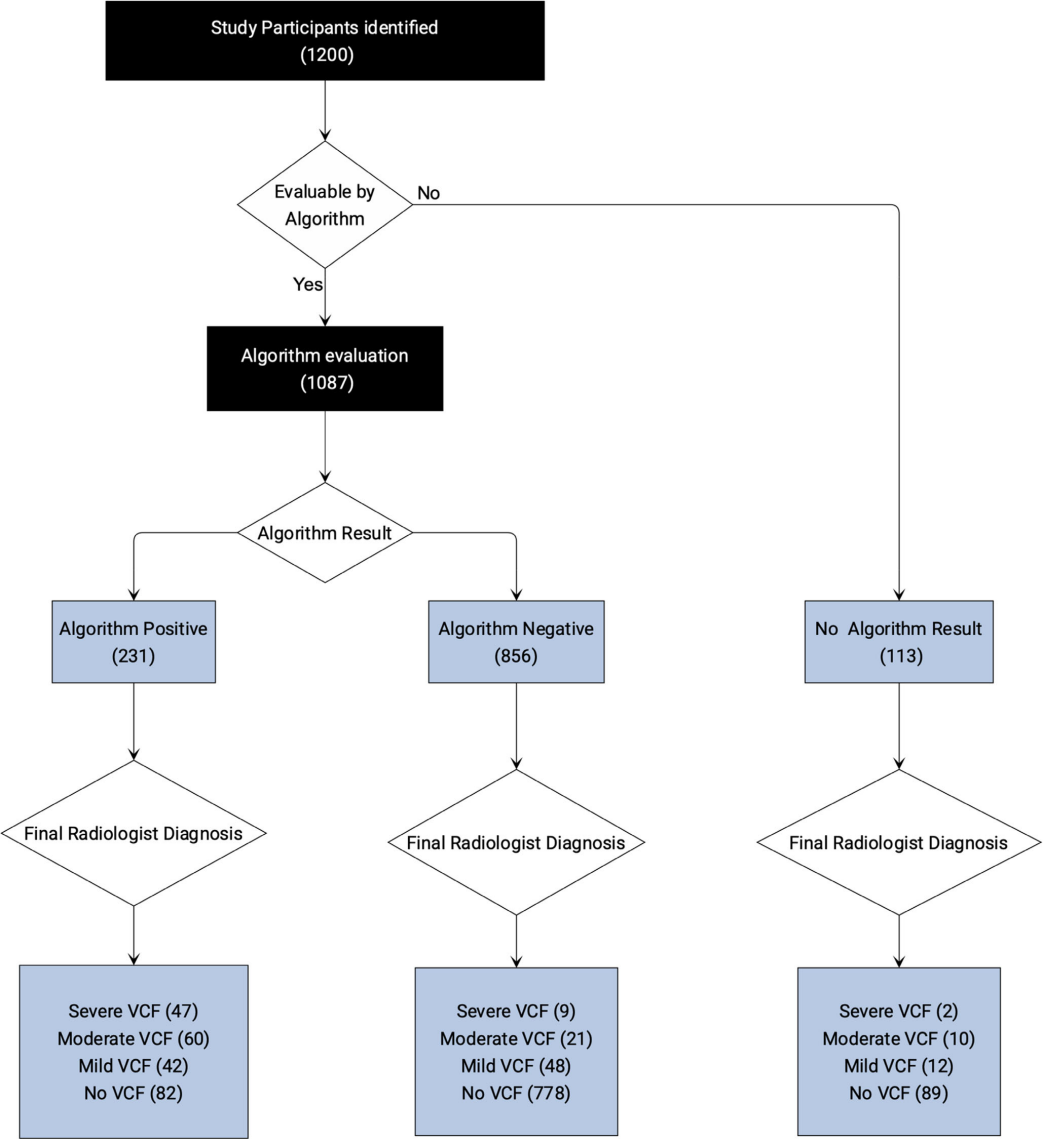
Study design flow diagram showing attrition and outcomes of study participants, by results of the vertebral compression fracture (VCF) algorithm.

**Fig. 2:**
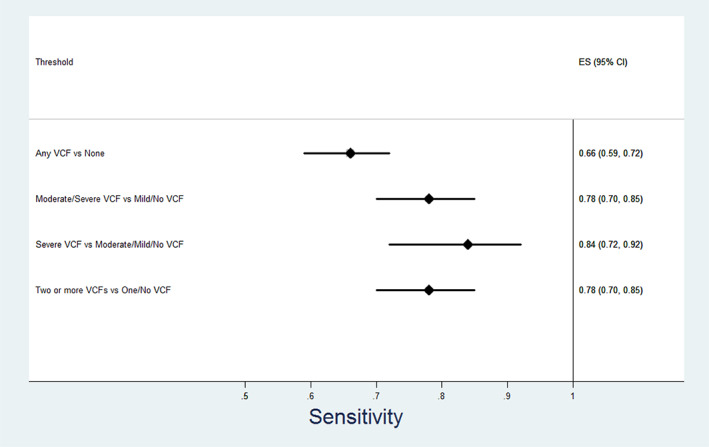
Performance of vertebral compression fracture (VCF) algorithm—sensitivity.

Of the 1200 study participants (962 chest CT, 169 abdomen/pelvis, and 69 for chest/abdomen/pelvis), the Zebra VCF algorithm was able to make a VCF determination on 1087 (90.6%, Figs. 1 and 2). The algorithm did not provide a reading for 113. Of the remaining 1087 study participants, 588 (54%) were women. Median age was 73 years (range 51–102; interquartile range 66–81) (Table 1).

**Table 1:** Patient Characteristics

Characteristic	No. (%)
Total patients	1200
Patients with non‐missing algorithm result	1087
Female	588 (49)
Age (years)	Median 73 (range 51–102)
Age group (years)	
50–60	154 (13)
61–70	311 (26)
71–80	345 (29)
81–90	215 (18)
91+	62 (5)
Missing sex/age	113 (9)

*Note*: The 588 women represented 54% of the patients with non‐missing algorithm result. 499 men were included among the 1087 (46%).

### Distribution of VCF

Based on radiologist diagnosis (reference standard), among the 1087 scans that were evaluable by the algorithm, 227 (21%) were determined to have at least one VCF. Ninety had mild VCF, 81 moderate VCF, and 56 severe VCF (Table 2). One hundred fifteen presented with two or more VCFs. After excluding patients with previously documented VCF, 161 of 996 (14%) were determined to have at least one VCF.

**Table 2:** Evaluation of CT Images for Vertebral Compression Fractures (VCF)

Characteristic	No. (%) algorithm‐evaluable (1087)
Algorithm diagnosis of VCF	231 (21)
Radiologist diagnosis of VCF	227 (21)
No. of fractures (patient level)	
No fracture	860 (79)
One fracture	112 (10)
Two or more fractures	115 (11)
Highest grade of fracture (patient level)	
No facture	860 (79)
Mild (Genant 1)	90 (8)
Moderate (Genant 2)	81 (7)
Severe (Genant 3)	56 (5)

### Performance characteristics

**Fig. 3:**
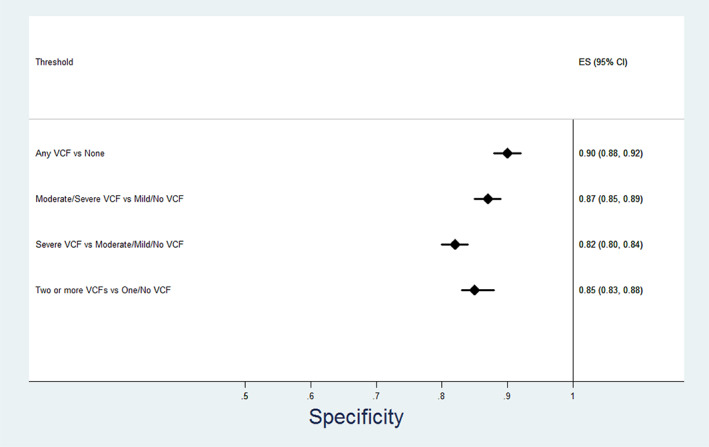
Performance of vertebral compression fracture (VCF) algorithm—specificity.

**Fig. 4:**
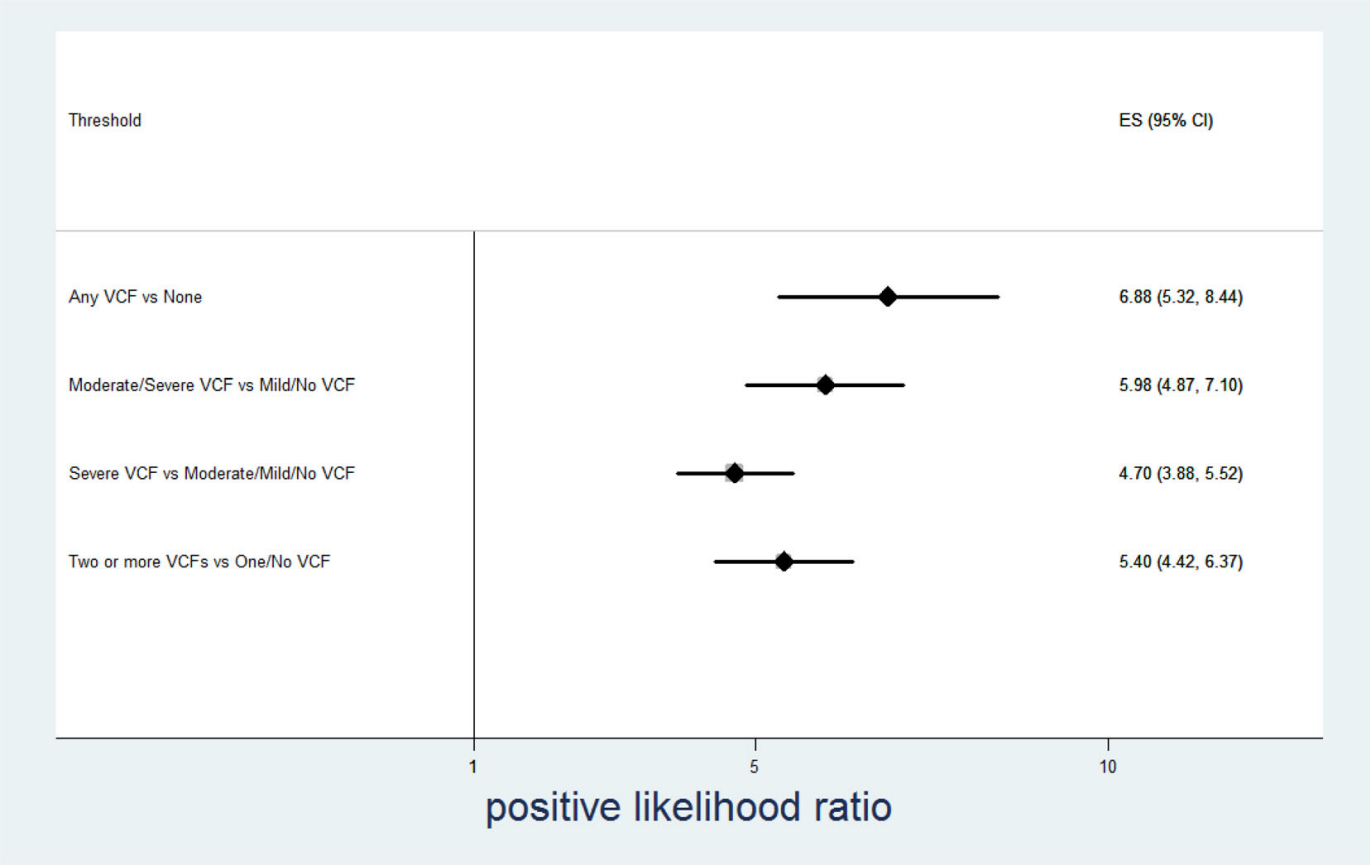
Performance of vertebral compression fracture (VCF) algorithm—positive likelihood ratio.

**Fig. 5:**
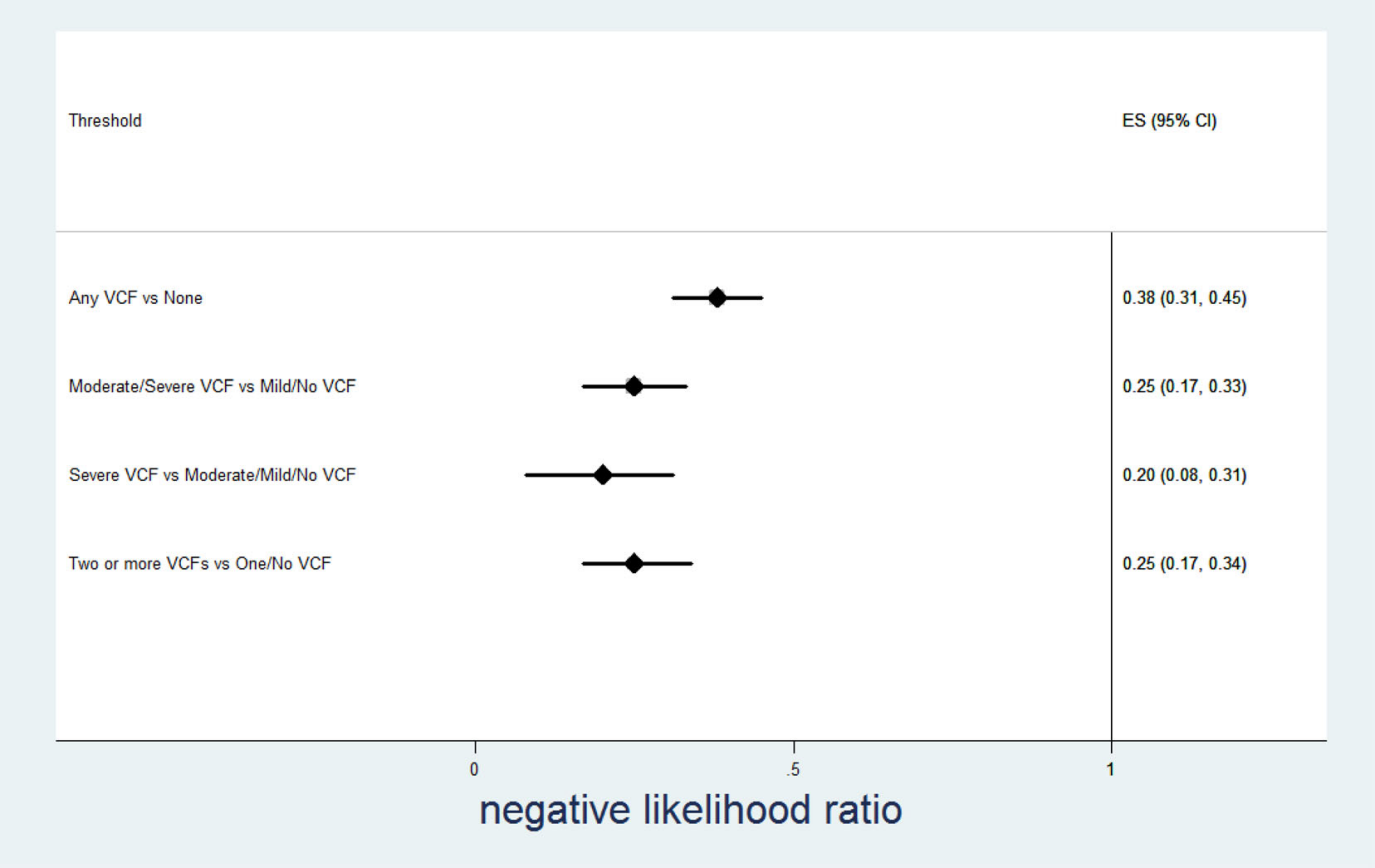
Performance of vertebral compression fracture (VCF) algorithm—negative likelihood ratio.

Table 3 and Figures 2, 3, 4, 5 show the relationship between radiologist diagnosis and the results from the Zebra VCF algorithm at the patient level. The sensitivity and specificity of the Zebra VCF algorithm in diagnosing any VCF were 0.66 (95% CI 0.59–0.72) and 0.90 (95% CI 0.88–0.92), respectively, and for diagnosing moderate/severe VCF were 0.78 (95% CI 0.70–0.85) and 0.87 (95% CI 0.85–0.89), respectively. In other words, a positive finding with the VCF algorithm was associated with 6.88 increased odds of having a VCF, relative to not having a VCF (95% CI 5.32–8.44) and 5.98 increased odds of having a moderate/severe VCF relative to not having such a fracture (95% CI 4.87–7.10).

**Table 3:** Performance of Vertebral Compression Fracture (VCF) Algorithm, Using the Radiologist Diagnosis as the Reference Standard

Radiologist diagnosis		VCF detection algorithm determination	
		Yes	No
Mild, moderate, or severe versus no VCF	Yes	149	78
	No	82	778
Moderate or severe versus mild or no VCF	Yes	107	30
	No	124	826
Severe versus mild, moderate, or no VCF	Yes	47	9
	No	184	847
Two or more fractures versus none/one VCF	Yes	90	25
	No	141	831

Most VCFs were identified at T_8_, T_11_‐L_2_ vertebral levels in the spine (Table 4).

**Table 4:** Sensitivity by Vertebral Level **(**Based on Radiologist Diagnosis at the Vertebral Level and Vertebral Compression Fracture [VCF] Algorithm [Algo] Diagnosis at the Patient Level)

	Any VCF			Moderate or severe VCF		
Vertebral level	Radiologist positive	Algo positive	Sensitivity (95% CI)	Radiologist positive	Algo positive	Sensitivity (95% CI)
T_1_	2	1	0.50 (0.01–0.99)	0		
T_2_	3	2	0.67 (0.09–0.99)	0		
T_3_	8	4	0.50 (0.16–0.84)	4	2	0.50 (0.07–0.93)
T_4_	9	5	0.56 (0.21–0.86)	3	3	1 (0.29–1)
T_5_	20	13	0.65 (0.41–0.85)	8	8	1 (0.63–1)
T_6_	25	17	0.68 (0.47–0.85)	10	7	0.70 (0.35–0.93)
T_7_	29	20	0.69 (0.49–0.85)	14	12	0.86 (0.57–0.98)
T_8_	50	32	0.64 (0.49–0.77)	24	17	0.71 (0.49–0.87)
T_9_	32	25	0.78 (0.60–0.91)	15	12	0.80 (0.52–0.96)
T_10_	34	28	0.82 (0.65–0.93)	10	10	1 (0.69–1)
T_11_	62	46	0.74 (0.62–0.84)	27	25	0.93 (0.76–0.99)
T_12_	70	52	0.74 (0.62–0.84)	48	38	0.79 (0.65–0.90)
L_1_	64	53	0.83 (0.71–0.91)	35	30	0.86 (0.70–0.95)
L_2_	35	31	0.88 (0.73–0.97)	22	19	0.86 (0.65–0.97)
L_3_	19	15	0.79 (0.54–0.94)	10	9	0.90 (0.55–1)
L_4_	12	10	0.83 (0.52–0.98)	6	6	1 (0.54–1)
L_5_	8	5	0.63 (0.24–0.91)	2	2	1 (0.16–1)

*Note*: 95% confidence intervals (CI) for the sensitivity were calculated using an exact binomial distribution.

At a patient level, it appeared that the Zebra VCF algorithm was able to identify most fractures that were located at T_5_ or below (Table 4).

### Interrater reliability of radiologists' measurements

The intraclass correlation coefficient as a measure of inter−radiologist reliability for determining the presence versus absence of at least one VCF among the 1200 sets of CT scans evaluated was 0.68 (95% CI 0.63–0.72). The corresponding metric for determining VCF severity for the most severe VCF for each study participant was 0.86 (95% CI 0.83–0.89) and for the number of VCFs was 0.80 (0.75–0.84), respectively.

## Discussion

The Zebra VCF algorithm works to identify approximately five‐sevenths (inclusive of non‐evaluable images) of moderate to severe VCF identifiable on CT studies of thorax or abdomen in adults, aged 50 years and older, who receive CT scans for other reasons, while falsely labeling a tenth of patients without fracture as having fracture. The sensitivity for all VCF was 0.66 of evaluable images (~59% of all images). The positive likelihood ratios of 6.9 and 6.0 for any fracture and for moderate to severe fracture versus other, respectively, means that a positive finding by the algorithm increases the odds of any VCF by almost 7× and for moderate to severe VCF by approximately 6×.

Kolanu and colleagues,^(^ ^19^ ^)^ who evaluated the performance of the Zebra VCF algorithm in 1686 thoracic/abdominal CT studies at an Australian single tertiary‐care facility, found that the algorithm had a lower sensitivity of 0.54 but a slightly higher specificity of 0.92 for all VCF. They did find sensitivity and specificity of 0.65 and 0.92, respectively, for moderate/severe (Genant 2/3) VCF. The study, however, had a major limitation in that CT scans were reviewed by a second radiologist only in situations where there were discrepancies between the VCF algorithm and the initial radiologist. This version of reference standard is non‐ideal in that the standard is much more dependent on the performance of one radiologist relative to others and can bias the apparent performance of the algorithm in ways that are difficult to generalize. In a previous study^(^ ^13^ ^)^ that reported on the training of the Zebra VCF algorithm, the authors evaluated the algorithm in a validation set, reportedly balanced between positive and negative samples, distinct from that used for training, and reported accuracy of 0.89, with sensitivity of 0.83 and specificity of 0.94. However, the authors did not report on procedures used for blinding, if any. Two other studies^(^ ^20^, ^21^ ^)^ evaluated the Zebra VCF algorithm in terms of feasibility and prediction, but neither of the studies was blinded or involved a systematic validation. Roux and colleagues^(^ ^21^ ^)^ reported on the use of the algorithm in a large cohort of French patients, but no validation by radiologists was done. Dagan and colleagues^(^ ^20^ ^)^ reported on the predictive performance of a Zebra‐defined CT scan‐based algorithm performed on scans taken before 2012 to predict major osteoporotic fractures between 2012 and 2017. Other studies^(^ ^22^, ^23^, ^24^, ^25^ ^)^ have evaluated other machine learning–based algorithms to identify VCFs in thoracic/abdominal CT scans performed for other reasons and most have reported very high sensitivity. However, these studies were limited by one or more of inadequate reference standard, inadequate blinding, or small study size.

Using routine CT scans of the chest or abdomen have some limitations in that they do not allow evaluation of the entire spine. Thoracic CT allows examination of the thoracic and upper lumbar spine.^(^ ^26^ ^)^ Abdominal CT allows examination of the lower thoracic (T_10_ and below) and lumbar spine.^(^ ^27^, ^28^ ^)^ The most frequent sites for VCFs are midthoracic (T_8_) and at the thoracolumbar junction (T_12_‐L_1_).^(^ ^29^, ^30^ ^)^ Therefore, most VCFs may be identified by CT scans of thorax or abdomen, but some will likely be missed. Additionally, a dedicated CT of the spine, either the entire thoracolumbar spine or the individual thoracic/lumbar spine, can be acquired with a more magnified view of the spine only, and the remainder of the soft tissues of the chest, abdomen, and pelvis are excluded from the scans. This allows a more detailed, focused evaluation of the spine.

Accurate diagnosis of VCF requires knowledge of other deformities that can generate false positives, including intervertebral osteochrondrosis, Schmorl's nodes, and congenital abnormalities.^(^ ^9^, ^26^ ^)^ These complicate the widespread implementation of computer‐aided diagnostic methods like the Zebra algorithm. As many of these entities may cause the appearances of the individual vertebral bodies to mimic various severities of VCFs, proper implementation will require validation of findings by trained or experienced medical staff.

Strengths of this research include the design, which ensured that radiologists were blind to the ratings of each other and to the ratings of the VCF algorithm. Further, the implementation of the VCF algorithm was implemented in such a way that it was blind to the rating of the radiologists. The data resulting from the two evaluations were handled by separate institutions and were only merged after the evaluations were complete. The study was also designed to have adequate sample size to estimate the performance metrics with reasonable precision. We made a valiant attempt to optimize the reference standard by using verification by expert neuroradiologists who regularly review spine CT scans as part of their regular work and resolving disagreements by a third neuroradiologist. However, there was some disagreement between radiologists, particularly with respect to determining severity (Genant grade) and to determining the vertebral level of the VCF. Although radiologists graded VCF at the level of the vertebra, the Zebra algorithm determined VCF at the patient level only.

The study had other limitations. We evaluated CT scans from the past rather than a prospective identification of patients. One of the consequences of this was that other than age and sex, we had very little covariate data on included patients and we did not know the reason for the examination. Furthermore, although reformats can be made of the thoracic or lumbar spine from the CT scans of the chest, abdomen, or pelvis, being that scans were identified retrospectively, the reformats were often made from the available data set (often reformatted at 2.5‐mm intervals) as opposed to the original volumetric thin data sets that are not set to the PACS (picture archiving and communication system) database due to storage limitations. This limits the resolution of the reformatted images that are used to determine the presence of VCFs. This issue would have been avoided in a fully prospective study. In addition, the implementation team had not included the 100 CT studies with previously defined verifiable VCF in the original data evaluated by the algorithm. These data were added later together with an additional 100 CT studies of undetermined VCF status, which were shuffled to maintain blinding. Further, the VCF algorithm was not able to provide a determination on almost 10% of CT images evaluated.

The Zebra VCF algorithm works to identify just over 70% of moderate to severe VCF in adults, aged 50 years and older, who receive CT scans for other reasons, provided evaluation can be done. Implementing the Zebra VCF algorithm within radiology systems may help to identify patients at increased fracture risk and could support the diagnosis of osteoporosis. When used as a step in a comprehensive diagnostic process, the Zebra computer‐aided diagnostic algorithm for VCF identification may be a helpful tool.

## Author Contributions

**John H Page:** Conceptualization; formal analysis; investigation; methodology; supervision; writing – original draft; writing – review and editing. **Franklin G Moser:** Conceptualization; investigation; validation; writing – review and editing. **Marcel M Maya:** Validation; writing – review and editing. **Ravi Prasad:** Validation; writing – review and editing. **Barry D Pressman:** Conceptualization; investigation; supervision; validation; writing – review and editing.

## Disclosures

The authors declare no competing non‐financial interests but the following competing financial interests: JHP is employed by and own shares of stock in Amgen Inc.; FGM, MMM, RP, and BDP received funding from Amgen Inc.

### Peer Review

The peer review history for this article is available at https://www.webofscience.com/api/gateway/wos/peer-review/10.1002/jbm4.10778.
